# Long-term impact of anastomotic leakage following esophageal atresia repair: feeding outcomes, esophageal morbidity, and reinterventions

**DOI:** 10.3389/fped.2026.1907146

**Published:** 2026-07-20

**Authors:** Suhyeon Ha, Hyunhee Kwon, Soo-Min Jung, Jung-Man Namgoong, Yong Jae Kwon

**Affiliations:** Division of Pediatric Surgery, Asan Medical Center Children's Hospital, Asan Medical Center, University of Ulsan College of Medicine, Seoul, Republic of Korea

**Keywords:** anastomotic leakage, anastomotic stricture, esophageal atresia, feeding outcomes, gastroesophageal reflux disease

## Abstract

**Purpose:**

Anastomotic leakage remains one of the most common complications after esophageal atresia (EA) repair. However, its impact on long-term esophageal morbidity and functional outcomes remains incompletely understood. This study evaluated the clinical consequences of postoperative anastomotic leakage following EA repair.

**Methods:**

We retrospectively reviewed patients with EA who underwent surgical repair at a single tertiary center between 2010 and 2024. Patients with type E EA were excluded. Clinical characteristics, operative variables, postoperative complications, feeding outcomes, and long-term esophageal morbidities were compared according to the presence of anastomotic leakage. Multivariable logistic regression analyses were performed to identify factors associated with anastomotic stricture and gastroesophageal reflux disease (GERD).

**Results:**

A total of 143 patients were included, of whom 36 (25.2%) developed anastomotic leakage. Patients with leakage had a higher incidence of anastomotic stricture than those without leakage (78% vs. 57%, *p* = 0.029). Leakage was also associated with delayed feeding progression, including longer times to feeding initiation (11 vs. 8 days, *p* = 0.022), oral feeding initiation (38 vs. 14 days, *p* = 0.005), achievement of full feeding (35 vs. 21 days, *p* = 0.037), and oral full feeding (58 vs. 26 days, *p* = 0.012). Unplanned reoperations were more frequent in the leakage group (31% vs. 11%, *p* = 0.008). In multivariable analysis, greater gap distance was independently associated with an increased risk of anastomotic stricture (adjusted OR 1.38, 95% CI 1.05–1.87, *p* = 0.026), whereas anastomotic leakage remained associated with GERD after adjustment for the available covariates (adjusted OR 2.60, 95% CI 1.14–6.26, *p* = 0.027).

**Conclusion:**

Anastomotic leakage after EA repair is associated with delayed feeding progression, increased esophageal morbidity, and a higher risk of unplanned reoperation. In addition, anastomotic leakage remained associated with GERD after adjustment for the available covariates. These findings suggest that patients who experience postoperative leakage represent a high-risk group that may benefit from careful long-term surveillance and multidisciplinary follow-up.

## 1 Introduction

Esophageal atresia (EA) is a rare congenital anomaly that requires surgical reconstruction during the neonatal period (1, 2). Despite substantial advances in surgical techniques and perioperative care, postoperative complications remain common and contribute significantly to long-term morbidity (3–5). Among these complications, anastomotic leakage is one of the most frequent adverse events following primary EA repair (5–7).

Although anastomotic leakage is generally regarded as an early postoperative complication, its clinical consequences may extend beyond the immediate postoperative period. Previous studies have suggested associations between leakage and subsequent esophageal morbidity, particularly anastomotic stricture formation (8–10). However, evidence regarding the long-term functional impact of leakage remains limited. In particular, the relationship between postoperative leakage and feeding progression, oral feeding achievement, and gastroesophageal reflux disease (GERD) has not been fully elucidated.

Functional feeding outcomes are especially important in patients with EA, as feeding difficulties may persist throughout childhood and substantially affect growth, development, and quality of life (11, 12). Delayed oral feeding acquisition and persistent reflux-related symptoms often require repeated interventions and prolonged follow-up (13). Therefore, understanding the long-term implications of postoperative leakage is essential for risk stratification and postoperative management.

The aim of this study was to evaluate the clinical impact of anastomotic leakage after EA repair. We compared feeding outcomes, esophageal morbidity, and reintervention rates between patients with and without leakage and investigated factors associated with anastomotic stricture and GERD.

## 2 Methods

### 2.1 Study design and patients

**Figure 1:**
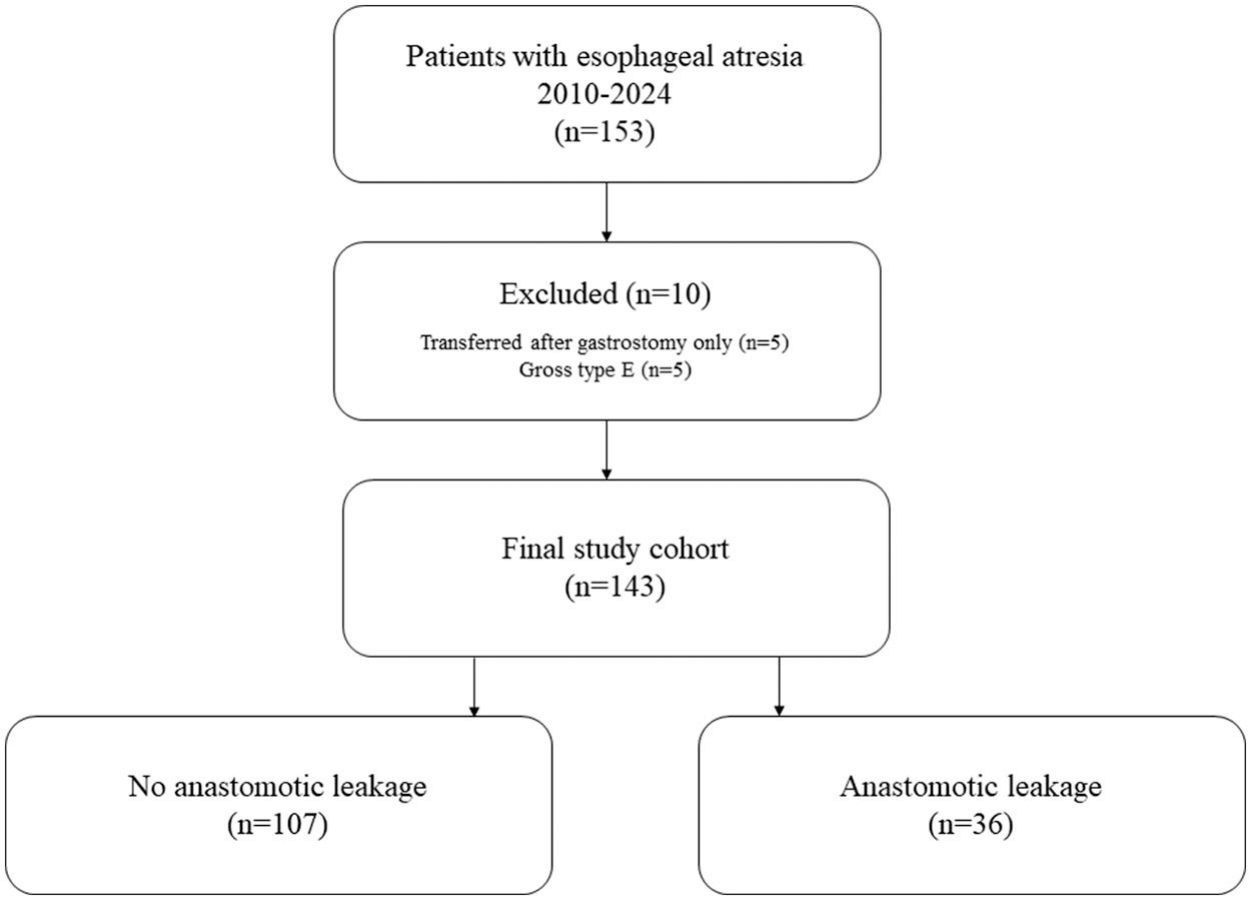
Flow diagram of patient selection and study cohort formation. Among 153 patients with esophageal atresia treated between 2010 and 2024, 10 patients were excluded (5 transferred after gastrostomy alone and 5 with Gross type E EA). The final cohort comprised 143 patients, including 107 without anastomotic leakage and 36 with anastomotic leakage.

This retrospective cohort study was conducted at a single tertiary referral center and included patients who underwent surgical repair for EA between January 2010 and December 2024. A total of 153 patients with EA were identified from the institutional database. Five patients who underwent gastrostomy alone and were subsequently transferred to other institutions, as well as five patients with isolated tracheoesophageal fistula without esophageal discontinuity (Gross type E), were excluded. Patients in these groups do not undergo esophageal anastomosis and therefore are not at risk for anastomotic leakage or anastomotic stricture. The remaining 143 patients were included in the analysis (Figure 1).

Demographic, perioperative, and postoperative data were collected from electronic medical records. Associated anomalies were defined as any congenital anomaly identified before or during follow-up and included cardiac, gastrointestinal, anorectal, vertebral, renal/genitourinary, limb, airway, craniofacial, neurologic, and other congenital abnormalities. Patients were categorized according to the presence or absence of postoperative anastomotic leakage. Baseline characteristics, operative variables, and postoperative outcomes were compared between groups.

### 2.2 Surgical and postoperative management

A transanastomotic feeding tube and a chest drain were routinely placed at the time of esophageal anastomosis. Patients were kept nil per os (NPO) until routine contrast esophagography, which was performed on postoperative day 7 to assess anastomotic integrity. In the absence of anastomotic leakage, the transanastomotic tube was removed, and oral feeding was initiated with small amounts of sterile water. If tolerated, oral intake was gradually advanced to breast milk or formula with progressive increases in volume until full oral feeding was achieved.

Patients with radiologically confirmed anastomotic leakage were managed according to institutional practice. Conservative management was the preferred approach for patients without major anastomotic disruption or recurrent tracheoesophageal fistula. In patients with a gastrostomy, enteral feeding through the gastrostomy was maintained while oral feeding was withheld until repeat contrast esophagography, routinely performed on postoperative day 14, confirmed improvement or resolution of the leakage. In patients who also had significant gastroesophageal reflux limiting gastric feeding, enteral nutrition was maintained via a transpyloric feeding tube placed through the gastrostomy or by jejunostomy when clinically indicated. In patients without a gastrostomy, fasting was continued until radiographic resolution of the leakage was confirmed. During this period, patients received supportive care including intravenous antibiotics and maintenance of chest drainage as clinically indicated. Reoperation was reserved for patients with major anastomotic disruption, recurrent tracheoesophageal fistula, or persistent leakage associated with clinical deterioration.

### 2.3 Exposure, definitions and outcomes

The exposure of interest was postoperative anastomotic leakage. Anastomotic leakage was defined as radiologic evidence of contrast extravasation at the anastomotic site on routine postoperative esophagography, which was typically performed on postoperative day 7.

Gap distance was obtained from the operative records and recorded as the distance between the proximal and distal esophageal ends at the time of esophageal anastomosis. Conventional long-gap esophageal atresia was defined as a gap length of at least two vertebral bodies between the proximal and distal esophageal segments, consistent with previous reports (14, 15). Anastomotic stricture was defined as a clinically significant narrowing at the anastomotic site diagnosed based on radiographic findings and/or clinical symptoms according to the treating surgeon's assessment. Endoscopic balloon dilation was performed according to each surgeon's clinical practice and was therefore analyzed separately as a treatment variable rather than as part of the definition of stricture.

Patients were categorized into leakage and no-leakage groups. Postoperative outcomes included anastomotic stricture, GERD, GERD-related treatment, feeding progression, unplanned reoperation, and postoperative recovery parameters. Feeding progression was assessed using the time to initiation of enteral feeding, time to initiation of oral feeding, time to achievement of full enteral feeding, and time to achievement of oral full feeding. Feeding-related outcomes were calculated as the number of days from primary esophageal repair to feeding initiation, oral feeding initiation, achievement of full feeding, and achievement of oral full feeding. GERD was defined as radiographic evidence of gastroesophageal reflux on esophagography in conjunction with compatible clinical symptoms, including nausea, vomiting, feeding intolerance, or regurgitation. Although this definition was consistently applied throughout the study, the diagnosis and management of GERD were based on routine clinical practice rather than a standardized institutional protocol.

### 2.4 Statistical analysis

Continuous variables are presented as median with interquartile range (IQR), and categorical variables as number and percentage. Baseline characteristics, operative variables, and postoperative outcomes were compared between patients with and without anastomotic leakage. Continuous variables were analyzed using the Wilcoxon rank-sum test, while categorical variables were compared using the chi-square test or Fisher's exact test, as appropriate.

To evaluate factors associated with postoperative outcomes, multivariable logistic regression analyses were performed using clinically relevant covariates selected *a priori*. Separate multivariable models were constructed for anastomotic stricture and GERD. The relationship between anastomotic leakage, stricture, and GERD was further explored using these models.

Odds ratios (ORs) and 95% confidence intervals (CIs) were calculated. Cases with missing data were excluded from the relevant analyses. All statistical tests were two-sided, and a *P* value < 0.05 was considered statistically significant. Statistical analyses were performed using R software (version 4.3.5; R Foundation for Statistical Computing, Vienna, Austria).

## 3 Results

### 3.1 Patient characteristics

A total of 143 patients were included, of whom 36 (25.2%) developed anastomotic leakage. Baseline characteristics according to leakage status are shown in Table 1. Patients with leakage had a lower prevalence of associated anomalies than those without leakage (69% vs. 92%, *p* = 0.004). Cardiac anomalies were the most common associated anomalies, followed by renal/genitourinary, anorectal, vertebral, and limb anomalies. The distribution of Gross classification differed between groups (*p* = 0.002), although type C was predominant in both groups. No significant differences were observed in sex, birth weight, prematurity, age at operation, VACTERL association, conventional long-gap EA status, or gap distance.

**Table 1:** Baseline characteristics according to leakage after esophageal atresia repair.

Variables	No leakage (*N* = 107)	Leakage (*N* = 36)	*P* value
Male sex	41 (38%)	19 (53%)	0.17
Birth weight, g	2,450 (2,050–2,840)	2,575 (2,092–2,920)	0.31
Prematurity	46 (43%)	16 (46%)	0.85
Age at operation, day	3 (2.0–6.0)	4 (2.0–8.0)	0.38
Associated anomalies	97 (92%)	25 (69%)	**0.004**
VACTERL association	54 (51%)	20 (57%)	0.7
Gross classification			**0.002**
A	15 (14%)	7 (19%)	
B	0 (0%)	1 (2.8%)	
C	92 (85.9%)	28 (78%)	
Long-gap esophageal atresia (≥2 vertebral bodies)	31 (29%)	11 (31%)	0.84
Gap distance, mm	20.0 (14.0–29.0)	19.5 (15.0–34.0)	0.66

Continuous variables are presented as median (interquartile range) and categorical variables as number (%).

*P* values were calculated using the Wilcoxon rank-sum test, Pearson's chi-square test, or Fisher's exact test, as appropriate.

Bold values indicate statistical significance (*p* < 0.05).

### 3.2 Operative characteristics according to anastomotic leakage

Operative characteristics are summarized in Table 2. There were no significant differences between the leakage and no-leakage groups with respect to repair strategy (primary vs. delayed repair), operative approach, tracheoesophageal fistula ligation method, or anastomotic suture material.

**Table 2:** Operative characteristics according to anastomotic leakage.

Variables	No leakage (*N* = 107)	Leakage (*N* = 36)	*P* value
Repair strategy			0.40
Primary repair	78 (73%)	23 (64%)	
Delayed repair	29 (27%)	13 (36%)	
Operative approach			0.25
Thoracotomy	57 (54%)	15 (43%)	
Thoracoscopy	48 (46%)	20 (57%)	
Tracheoesophageal fistula ligation method			0.59
Hem-o-lok	5 (5.6%)	3 (10%)	
Suture	71 (80%)	22 (73%)	
Hem-o-lok and suture	13 (15%)	5 (17%)	
Anastomotic suture material			0.50
Vicryl	18 (19%)	7 (23%)	
PDS	64 (66%)	16 (53%)	
Vicryl and PDS	14 (14%)	7 (23%)	
Others	1 (1%)	0 (0%)	

Data are presented as number (%). *P* values were calculated using Fisher's exact test.

Patients with Gross type A esophageal atresia were excluded from analyses of tracheoesophageal fistula ligation. Denominators may vary because of missing operative records in transferred patients or incomplete documentation.

### 3.3 Postoperative outcomes according to anastomotic leakage

**Figure 2:**
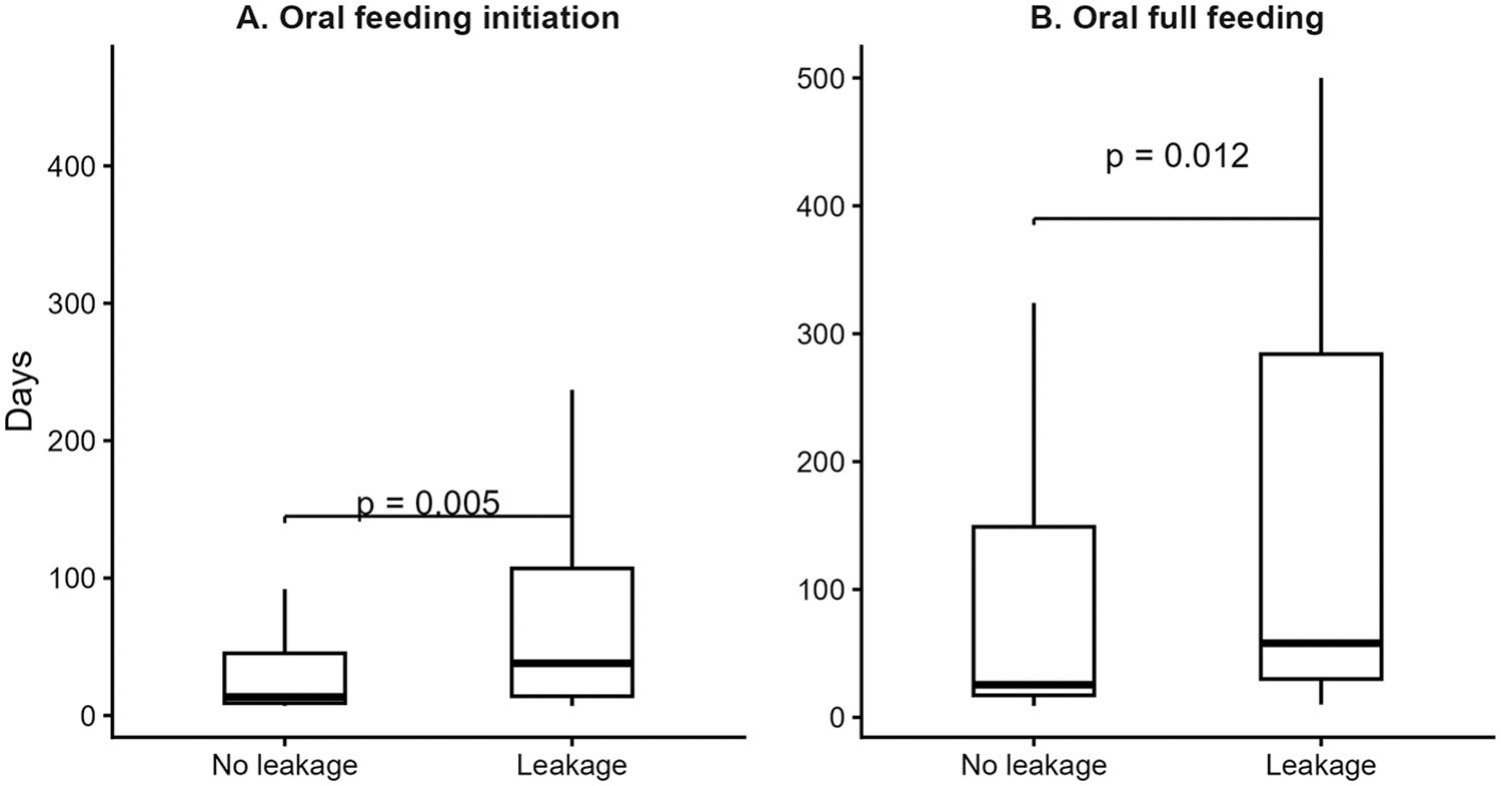
Oral feeding progression according to anastomotic leakage after esophageal atresia repair. Boxplots compare the time to oral feeding initiation **(A)** and the time to achievement of oral full feeding **(B)** between patients with and without anastomotic leakage. The central line represents the median, boxes indicate the interquartile range (IQR), and whiskers represent 1.5  ×  IQR. Individual dots represent individual patients. Patients with anastomotic leakage experienced significantly delayed oral feeding initiation (median 38 vs. 14 days, *p* = 0.005) and achievement of oral full feeding (median 58 vs. 26 days, *p* = 0.012). For visualization purposes, values exceeding 600 days were truncated at 600 days in panel B.

Postoperative outcomes according to anastomotic leakage status are summarized in Table 3. Patients who developed anastomotic leakage had a significantly higher incidence of anastomotic stricture compared with those without leakage (78% vs. 57%, *p* = 0.029). The leakage group also experienced delayed feeding progression, including a longer time to feeding initiation (11 vs. 8 days, *p* = 0.022), oral feeding initiation (38 vs. 14 days, *p* = 0.005), achievement of full feeding (35 vs. 21 days, *p* = 0.037), and oral full feeding (58 vs. 26 days, *p* = 0.012) (Figure 2).

**Table 3:** Postoperative outcomes according to anastomotic leakage.

Variables	No leakage (*N* = 107)	Leakage (*N* = 36)	*P* value
Anastomosis stenosis	60 (57%)	28 (78%)	**0.029**
Number of balloon dilatations	0.0 (0.0–2.0)	1.0 (0.0–3.5)	0.16
GERD	48 (45%)	23 (64%)	0.082
GERD operation	6 (5.6%)	4 (11%)	0.26
Time to feeding start, days	8 (6–12)	11 (7–26)	**0.022**
Time to oral feeding start, days	14 (9–47)	38 (14–107)	**0.005**
Time to full feeding, days	21 (15–37)	35 (19–48)	**0.037**
Time to oral full feeding, days	26 (17–149)	58 (30–284)	**0.012**
Unplanned reoperation	12 (11%)	11 (31%)	**0.008**

Values are presented as median (interquartile range) for continuous variables and number (%) for categorical variables. *P* values were calculated using the Wilcoxon rank-sum test or Fisher's exact test, as appropriate. Missing values were excluded from each analysis.

Time to feeding start, oral feeding start, full feeding, and oral full feeding were calculated from the date of primary esophageal repair. Implausible negative intervals were treated as missing values.

GERD, gastroesophageal reflux disease.

Bold values indicate statistical significance (*p* < 0.05).

In addition, patients with leakage underwent unplanned reoperations more frequently than those without leakage (31% vs. 11%, *p* = 0.008). In the leakage group, most reoperations were performed for leakage-related complications, including persistent anastomotic leakage and recurrent tracheoesophageal fistula, whereas reoperations in the no-leakage group were more commonly related to anastomotic stricture or other postoperative complications. Although gastroesophageal reflux disease (GERD) was more common in the leakage group (64% vs. 45%), the difference did not reach statistical significance (*p* = 0.082). Similarly, no significant differences were observed in the number of balloon dilatations or the rate of anti-reflux surgery between groups.

At the final follow-up, 129 of 143 patients (90.2%) achieved full oral feeding. Fourteen patients did not achieve full oral feeding. Among these, 11 patients died before oral feeding could be established because of severe comorbid conditions, including heart failure, pulmonary hypertension, renal failure, and disseminated intravascular coagulation. The remaining three patients remained dependent on gastrostomy feeding and required prolonged ventilator support via tracheostomy.

### 3.4 Predictors of anastomotic stricture

Logistic regression analyses for anastomotic stricture are summarized in Table 4. In univariable analysis, both anastomotic leakage (OR 2.68, 95% CI 1.16–6.81, *p* = 0.027) and greater gap distance (OR 1.39, 95% CI 1.07–1.87, *p* = 0.020) were associated with an increased risk of anastomotic stricture. After adjustment for gap distance, prematurity, and associated anomalies, gap distance remained an independent predictor of stricture formation (adjusted OR 1.38, 95% CI 1.05–1.87, *p* = 0.026), whereas the association between anastomotic leakage and stricture was attenuated and did not reach statistical significance (adjusted OR 2.43, 95% CI 0.99–6.48, *p* = 0.061).

**Table 4:** Logistic regression analysis for anastomotic stricture.

Variables	Univariable OR(95% CI)	*P* value	Multivariable OR (95% CI)	*P* value
Anastomotic leakage	2.68 (1.16–6.81)	**0.027**	2.43 (0.99–6.48)	0.061
Gap distance, mm	1.39 (10.70–18.70)	**0.020**	1.38 (10.50–18.70)	**0.026**
Prematurity	0.93 (0.47–1.84)	0.823	0.87 (0.42–1.81)	0.717
Associated anomalies	0.49 (0.15–1.36)	0.193	0.84 (0.24–2.65)	0.777

Odds ratios (ORs) with 95% confidence intervals (CIs) were calculated using logistic regression analysis. Variables with *P* < 0.10 in univariable analysis were included in the multivariable model.

Bold values indicate statistical significance (*p* < 0.05).

### 3.5 Multivariable analyses for postoperative sequelae

Logistic regression analyses for GERD are summarized in Table 5. In univariable analysis, anastomotic leakage demonstrated a trend toward an association with GERD (OR 2.14, 95% CI 0.99–4.77, *p* = 0.056). After adjustment for gap distance, prematurity, and associated anomalies, anastomotic leakage remained associated with GERD after adjustment for the available covariates (adjusted OR 2.60, 95% CI 1.14–6.26, *p* = 0.027). No significant associations were observed for gap distance, prematurity, or associated anomalies.

**Table 5:** Logistic regression analysis for gastroesophageal reflux disease.

Variables	Univariable OR (95% CI)	*P* value	Multivariable OR (95% CI)	*P* value
Anastomotic leakage	2.14 (0.99–4.77)	0.056	2.60 (1.14–6.26)	**0.027**
Gap distance, mm	0.88 (6.90–11.11)	0.291	0.87 (6.80–11.12)	0.265
Prematurity	0.98 (0.50–1.90)	0.941	0.91 (0.45–1.82)	0.790
Associated anomalies	1.24 (0.48–3.29)	0.654	1.65 (0.57–5.09)	0.363

Odds ratios (ORs) with 95% confidence intervals (CIs) were calculated using logistic regression analysis. Variables with *P* < 0.10 in univariable analysis were included in the multivariable model.

Bold values indicate statistical significance (*p* < 0.05).

## 4 Discussion

Anastomotic leakage is traditionally regarded as one of the most important complications following esophageal atresia repair because its consequences may extend well beyond the immediate postoperative period (5, 16, 17). Leakage has been implicated in a cascade of postoperative events, including local inflammation, impaired anastomotic healing, stricture formation (6, 16), and subsequent esophageal dysfunction (18). These sequelae may ultimately contribute to feeding difficulties, recurrent interventions, and gastroesophageal reflux disease (GERD) (13, 19). However, despite the widespread assumption that leakage adversely affects long-term outcomes, evidence supporting its impact on functional recovery remains limited.

In this study, anastomotic leakage occurred in approximately one-quarter of patients undergoing repair of esophageal atresia and was associated with substantial postoperative morbidity. Patients with leakage experienced delayed feeding progression, higher rates of anastomotic stricture, and increased unplanned reoperation rates. Furthermore, anastomotic leakage remained associated with GERD after adjustment for the available covariates. However, this association should not be interpreted as evidence of a causal relationship. Anastomotic leakage may instead represent a surrogate marker of increased anastomotic tension, which is a recognized determinant of postoperative esophageal dysfunction. Although gap distance was included in the multivariable model, it is only a surrogate measure of operative tension and may not fully capture the technical complexity of the repair. Therefore, residual confounding by unmeasured anastomotic tension cannot be excluded. Nevertheless, these findings suggest that anastomotic leakage identifies a subgroup of patients at increased risk for adverse long-term functional outcomes and who may benefit from closer postoperative surveillance. These findings suggest that anastomotic leakage identifies a subgroup of patients at increased risk for adverse long-term functional outcomes who may benefit from closer postoperative surveillance. Our interpretation is supported by recent analyses from the EUPSA Registry, which suggest that anastomotic tension is a major determinant of both leakage and subsequent postoperative morbidity, and that anastomotic leakage may represent a marker of technically challenging repair rather than the direct cause of subsequent complications (20). Furthermore, recent registry data have demonstrated that baseline patient characteristics and associated anomalies substantially influence postoperative outcomes, reinforcing the need for cautious interpretation of associations observed in retrospective studies (21).

Previous studies have consistently identified anastomotic leakage as a risk factor for subsequent anastomotic stricture formation (22, 23). Leakage may induce local inflammation, tissue injury, and excessive scar formation during the healing process, thereby predisposing patients to luminal narrowing (24–26). In our cohort, patients with leakage demonstrated a significantly higher incidence of stricture than those without leakage. Although gap distance remained independently associated with stricture formation in multivariable analysis, anastomotic leakage showed a strong trend toward significance (adjusted OR 2.43, 95% CI 0.99–6.48, *p* = 0.061). Given the relatively small number of leakage events, the possibility of insufficient statistical power should be considered. Therefore, our findings suggest that both anastomotic tension, reflected by gap distance, and postoperative leakage may contribute to the development of stricture.

One of the most important findings of this study was the consistent delay in feeding progression among patients who experienced anastomotic leakage. Leakage was associated with prolonged times to feeding initiation, oral feeding initiation, achievement of full feeding, and achievement of oral full feeding. Although previous studies have largely focused on structural complications such as strictures (5, 27, 28), relatively little attention has been paid to functional feeding outcomes. Feeding difficulties represent a major source of long-term morbidity in patients with esophageal atresia and may adversely affect growth, development, and quality of life (11, 12). Our findings suggest that the consequences of leakage extend beyond anatomic healing and may substantially delay functional recovery of oral feeding.

Another notable finding was the independent association between anastomotic leakage and GERD. After adjustment for gap distance, prematurity, and associated anomalies, patients with leakage had more than twice the odds of developing GERD. The mechanism underlying this association remains uncertain but may involve impaired esophageal motility, altered esophageal compliance, and disruption of normal healing processes following leakage. While GERD is known to be common in patients with esophageal atresia, our results suggest that postoperative leakage may represent an additional risk factor for reflux-related morbidity.

This study has several strengths. To our knowledge, it represents one of the larger single-center studies specifically evaluating the clinical consequences of anastomotic leakage following EA repair. The relatively large cohort and consistent management within a specialized pediatric surgical center reduced variability in surgical and postoperative care. In addition, unlike many previous studies that focused primarily on structural complications such as anastomotic stricture, we comprehensively assessed functional outcomes, including feeding progression, oral feeding achievement, and GERD. These outcomes are highly relevant to patients and caregivers and provide a more comprehensive understanding of the long-term impact of anastomotic leakage.

This study has several limitations. First, its retrospective design may have introduced selection and information bias. Second, as a single-center study, the generalizability of our findings may be limited. Third, the diagnosis and management of GERD were based on clinical practice rather than a standardized protocol, which may have resulted in heterogeneity in outcome assessment. Fourth, the indications for endoscopic balloon dilation were not completely standardized among surgeons, reflecting differences in clinical practice that may have influenced the management of anastomotic stricture. Fifth, the severity of anastomotic leakage was not systematically classified, precluding analysis of whether the extent of leakage influenced long-term outcomes. Finally, although the fundamental surgical principles and postoperative management protocol remained largely unchanged throughout the study period, minimally invasive thoracoscopic repair was gradually adopted over time, and subtle changes in perioperative management and individual surgeon preferences may have influenced postoperative outcomes. Nevertheless, this study represents one of the larger single-center analyses focusing on the clinical consequences of anastomotic leakage after EA repair. The relatively large cohort, consistent surgical management within a specialized pediatric surgical center, and comprehensive evaluation of feeding-related outcomes strengthen the validity of our findings.

## 5 Conclusion

Anastomotic leakage following esophageal atresia repair is associated with delayed feeding progression, increased esophageal morbidity, and a higher rate of unplanned reoperation. In addition, anastomotic leakage remained associated with gastroesophageal reflux disease after adjustment for the available covariates. These findings suggest that postoperative anastomotic leakage identifies a subgroup of patients at increased risk for adverse long-term outcomes who may benefit from closer surveillance and multidisciplinary follow-up.

## Data Availability

The raw data supporting the conclusions of this article will be made available by the authors, without undue reservation.
